# The Relegated Goal of Health Institutions: Sexual and Reproductive Health

**DOI:** 10.3390/ijerph18041767

**Published:** 2021-02-11

**Authors:** Juan Miguel Martínez-Galiano, Miguel Delgado-Rodríguez

**Affiliations:** 1 Department of Nursing, University of Jaen, 23071 Jaén, Spain; 2 Consortium for Biomedical Research in Epidemiology and Public Health (CIBERESP), 28029 Madrid, Spain; mdelgado@ujaen.es; 3 Department of Health Sciences, University of Jaen, 23071 Jaén, Spain

Sexual and reproductive health does not always receive the attention it deserves and frequently is not supported with the necessary resources to guarantee its maintenance. The World Health Organization (WHO) is aware of its importance and in its structure has specific programs focused on this health aspect.

Although conceptually sexual and reproductive health is seen as a united whole, this does not occur in some parts of the world. Sexual health, according to the WHO, is a state of physical, mental, and social well-being concerning sexuality. It requires a positive and respectful approach to sexuality and sexual relations, as well as the possibility of having pleasant and safe sexual experiences that are free from all coercion, discrimination, and violence. Reproductive health focuses on all aspects of the reproductive system, its functions, and its processes. [1]

The WHO aims to give a joint approach to sexual and reproductive health with which it intends to support the development of research and learning policies in this field. Even the United Nations General Assembly has established the specific goal of guaranteeing universal access to sexual and reproductive health services by 2030.

One of the biggest challenges in sexual and reproductive health is sexually transmitted infections (STI). For the development of well-oriented health programs on this issue, it is necessary to know the attitude of health professionals towards patients with STIs. One of the most relevant STIs, due to the absence of curative treatments and vaccines, is human immunodeficiency virus (HIV); the results of a study carried out in Spain with nursing students show that they have a positive attitude towards HIV patients, and this may improve their future healthcare practice in tackling this condition [2]. The identification of groups vulnerable to STIs has a special relevance for prevention programs; Perez-Morente et al. have found that immigrant women in periods of crisis are more susceptible to acquiring an STI [3,4]. Screening and diagnostic methods, improved treatments, as well as the availability of more effective vaccines also deserve special attention. Alderete has identified a new *Trichomonas vaginalis* epitope chain protein and the epitope chain proteins of infectious agents that may have potential applicability for a possible vaccine against this parasite [5]. Health education plays a special role in the implementation of STI prevention programs, for which it is necessary to identify risky practices. In this issue, Santa-Barbara et al. show in their results that the practice of oral sex, the practice of anal sex, and the non-use of condoms are clear risk factors. [6] Valid instruments are available, as shown by Sánchez-Mendoza et al., to determine the self-efficacy of condom use in adolescents, [7] one of the social strata with the highest risk of acquiring STIs. This favours the design of adequate strategies designed to better promote the use of condoms as a method of protection against STIs.

Family planning is another basic component of sexual and reproductive health. Contraceptive methods allow people to enjoy sexual acts without the worry of unwanted pregnancy and help in family planning. Adolescents are the most susceptible group to unwanted pregnancies, and this occurs in people residing in higher-risk environments or more disadvantaged places, as shown by the results of Ávila-Burgos et al. in Mexico. [8] Accessibility to contraceptive methods is critical for providing the correct medication and giving adequate instructions to avoid unwanted pregnancies. This is displayed in this issue by Langer et al. in a study carried out in pharmacies in Germany. [9]. In the case of having to perform an abortion due to an unwanted pregnancy or for any other reason, it must be carried out in safe conditions for the woman, and therefore it is necessary to implement training and awareness programs [10]. Besides this, it is a priority to have adequate legislation to guarantee the safety of women. Legal restrictions on abortion can increase maternal and infant morbidity and mortality. This is what Pabayo et al. conclude in a study performed in the United States in which infant mortality in states with restrictive abortion laws are compared with that of states with more permissive laws [11].

Women are the main recipients of health programs on sexual and reproductive health. They may suffer from chronic diseases, such as endometriosis, which causes chronic fatigue, a worse quality of life, greater anxiety, and worse sleep, among other consequences, as Mundo-López et al. [12] show. On the other hand, clinical and educational practices during pregnancy and childbirth influence the health status of women. For example, Cano-Ibáñez et al. [13] show that correct nutrition and promoting a dietary pattern based on the Mediterranean diet improve the outcome of childbirth. Another example is provided by Infante-Torres et al. in a systematic review with meta-analysis [14] in which they show that care without prolonged expulsive periods, since an excessive duration of this labour stage leads to greater neonatal morbidity, improves newborns’ health. Postpartum post-traumatic stress disorder is an increasingly attention-grabbing disorder. For this reason, predictive models are useful, such as the one developed by Hernandez-Martinez et al. that helps to predict the risk of this disease [15]. Prenatal care is essential, but it is not practised in the same way in all areas or by all women. Thus, for example, in China women belonging to ethnic minorities and living in rural areas initiate pregnancy control later [16]. This necessitates the development of special programs for these situations. Not only must the woman be cared for, but her satisfaction with the healthcare process must also be taken into account. Instruments are available to gain knowledge of this aspect concerning attendance at delivery, such as the satisfaction scale with the assistance received, as validated by Pozo-Cano et al. [17].

Violence must be taken into account in reproductive health. The WHO in 2014 made a declaration for the prevention and eradication of disrespect and mistreatment during childbirth care in health centres; however, the figures of obstetric violence a few years later remain high [18]. Gender-based violence is a serious public health problem; it is exercised in various ways, with pregnancy being a clear risk factor. The umbrella review carried out by Roman-Galvez et al. [19] makes the magnitude of this problem visible and shows the high prevalence figures around the world. Another form of violence against women is female genital mutilation. Turkmani et al. present a conceptual model, based on cultural values and the physical and emotional needs of women, as a framework to guide maternity services to better address this problem [20]. The same authors conclude that empowering women and increasing their awareness of their health care rights can help involve women as active partners in the design and delivery of health information.

To progress in research and therefore in its application to the protection of sexual and reproductive health, it is necessary to take into account the social and health professionals, scientific societies, and institutions involved. García-Martin et al. stress the importance of cooperation between health professionals, academic and health institutions, and the community as an essential point to improve research quality and make significant advances in the field of women’s health [21].

Every health program needs to address popular beliefs that do not have any scientific proof. For instance, some Chinese women maintain a preference for male children, which can cause a greater number of pregnancies with their associated risks [22].

In summary, it is necessary to develop policies and strategies tackling sexual and reproductive health in collaboration with the different agents involved to address its different components, such as family planning, STIs, women’s health, pregnancy, and violence, among others. These policies must be translated into primary prevention, screening, early treatment, and rehabilitation programs. For the development of these programs, it is necessary to identify the most vulnerable populations, as well as their risk factors. They should also be based on scientific evidence and apply validated tools. Research in sexual and reproductive health should be promoted so that its results are the basis for the design of appropriate policies and strategies in sexual and reproductive health.

## Data Availability

The data used are available from the corresponding author on reasonable request.
